# WILLS, WEALTH, AND RACE

**DOI:** 10.1093/geroni/igae098.0969

**Published:** 2024-12-31

**Authors:** J P Aubry, Gal Wettstein, Alicia Munnell

**Affiliations:** Boston College, Chestnut Hill, Massachusetts, United States; Boston College, Chestnut Hill, Massachusetts, United States; Boston College, Chestnut Hill, Massachusetts, United States

## Abstract

Inheritances make up a substantial share of national wealth, but are often overlooked in discussions of retirement security. Racial gaps in inheritances are likely to exacerbate racial disparities in wealth. One reason that Black and Hispanic decedents are less likely to pass down meaningful estates is that they are far less likely to have a will than non-Hispanic Whites. This paper documents racial gaps in receiving an inheritance, in the likelihood of having a will, and in the expectation of leaving significant bequests. The analysis then looks at the relationship between bequest expectations and realized bequests to see whether expectations are a good predictor of real outcomes. The results show that Black and Hispanic decedents and those who die without a will are less likely to achieve their bequest expectations. This paper is the first of a three-part series. The second part will be a survey to see if a plausible intervention might increase the adoption of wills, whether writing of a will increases intended bequests, and, in conjunction with the estimates of this paper, whether wills would likely increase realized bequests. The third part of the project will examine the impact of will incentives on racial wealth gaps when compounded across multiple generations.

